# Supplementary material for: Revealing defective interfaces in perovskite solar cells from highly sensitive sub-bandgap photocurrent spectroscopy using optical cavities
Source: Nat Commun. 2022 Jan 17;13:349. doi: 10.1038/s41467-021-27560-6 (PMC8764070; doi:10.1038/s41467-021-27560-6)
Supplement: Supplementary file 1 — Supplementary Information [file 41467_2021_27560_MOESM1_ESM.pdf]

Supplementary information

Revealing defective interfaces in perovskite solar cells from highly sensitive sub-bandgap photocurrent spectroscopy using optical cavities

van Gorkom et al.

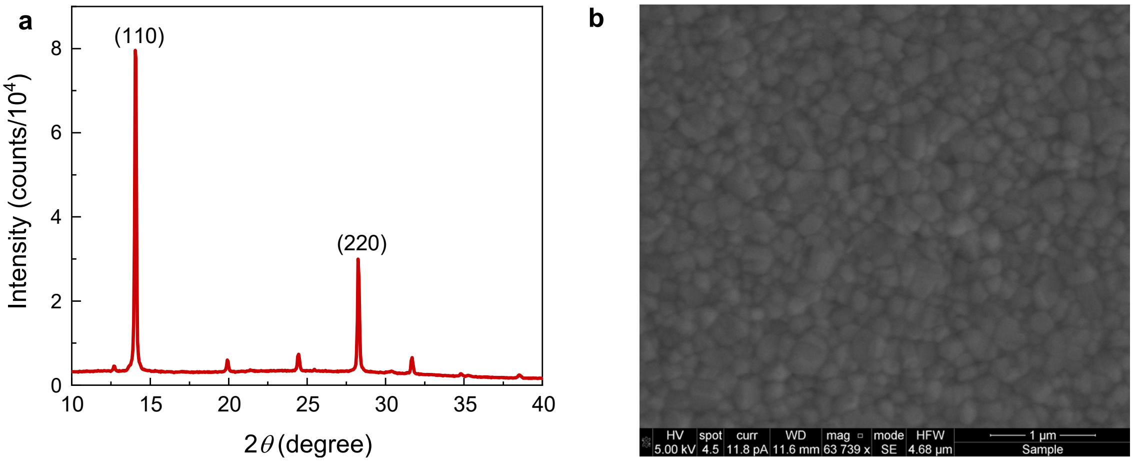

Supplementary Figure 1. Characterization of $FA_{0.67}MA_{0.33}PbI_{2.85}Br_{0.15}$ perovskite films. **a** XRD. **b** SEM image.

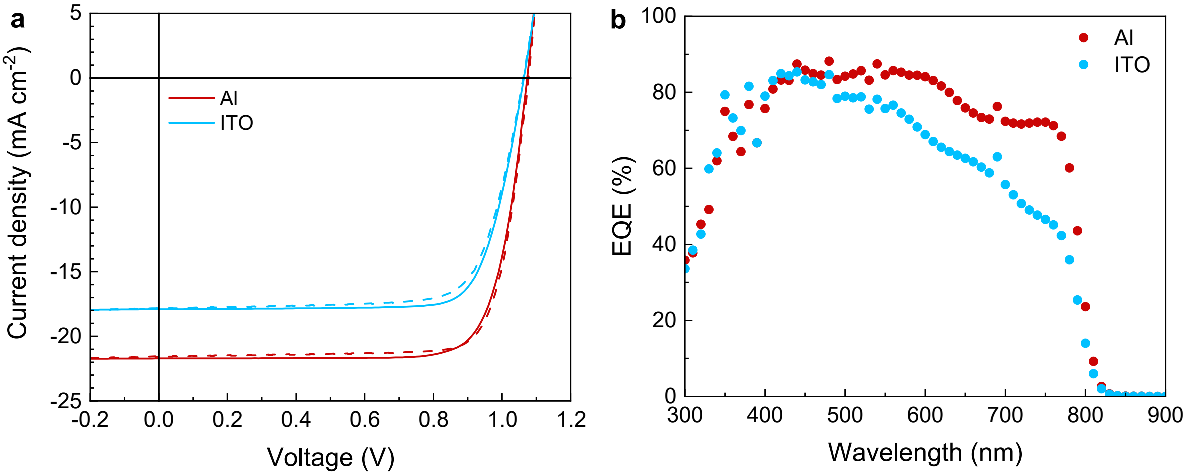

	Scan	J_{sc} (mA cm^{-2})	Integrated J_{sc} (mA cm^{-2})	V_{oc} (V)	FF	PCE (%)
Al	Reverse	21.9	21.1	1.06	0.77	17.9
	Forward	21.7		1.07	0.76	17.6
ITO	Reverse	17.9	17.9	1.06	0.78	14.7
	Forward	17.8		1.06	0.75	14.2

Supplementary Figure 2. ***J-V* characteristics, EQEs and photovoltaic parameters** of glass/ITO/PTAA/$FA_{0.67}MA_{0.33}PbI_{2.85}Br_{0.15}$/PCBM/AZO solar cells with Al and ITO back electrodes. **a** *J-V* characteristics with reverse and forward scan directions are shown in the solid and dashed lines, respectively. **b** EQEs. The EQE is integrated to give the integrated J_{sc}.

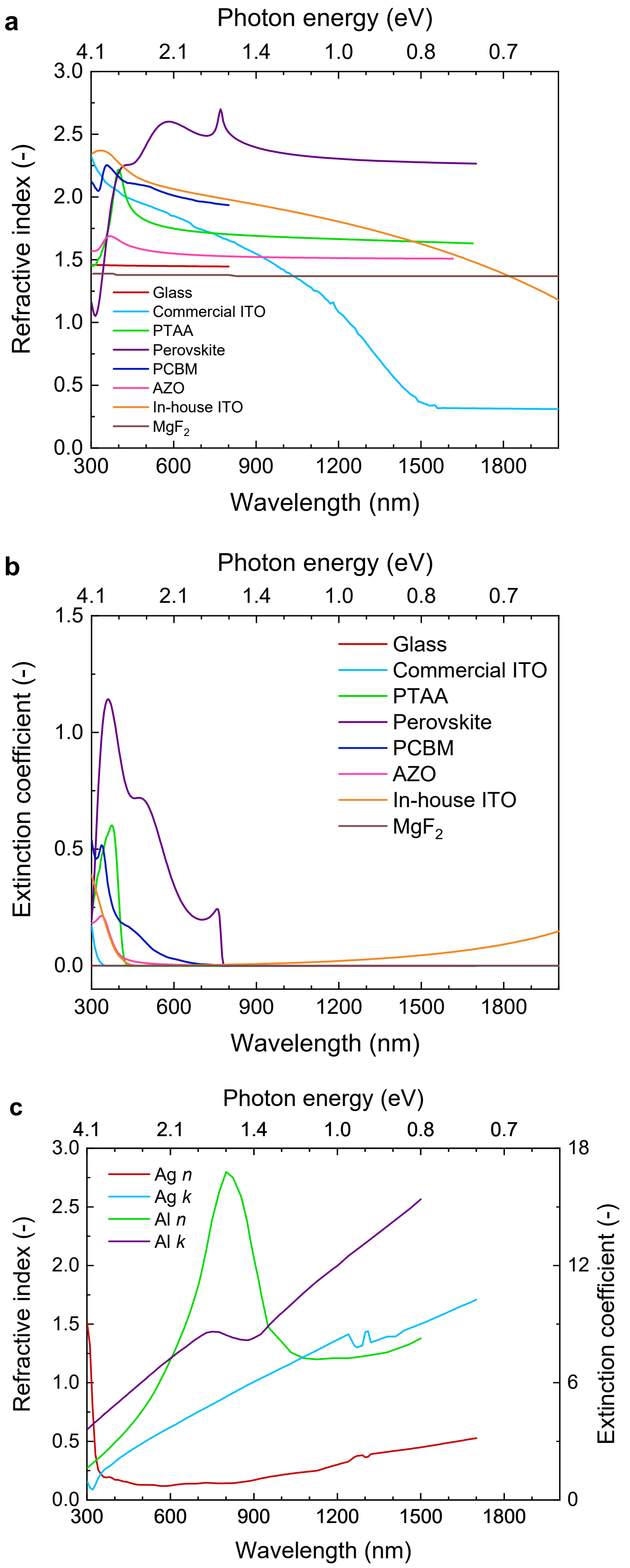

Supplementary Figure 3. Optical constants of materials used in the simulations of the electric field intensities. **a** Refractive indices. **b** Extinction coefficients. **c.** Refractive index and extinction coefficient of Ag and Al. The values are extrapolated where necessary.

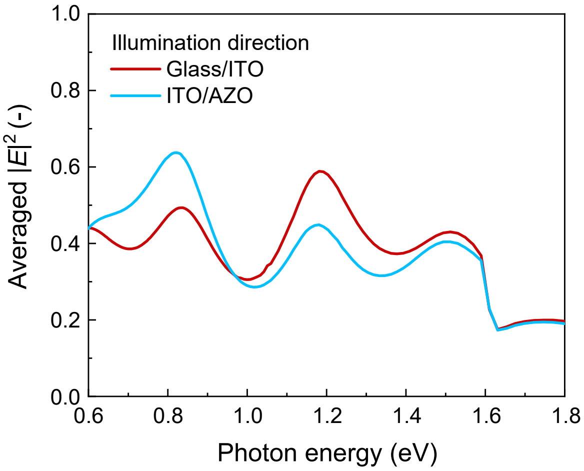

Supplementary Figure 4. Simulated optical electric field $|E|^2$ in a semitransparent p-i-n perovskite cell for a glass/ITO/PTAA/$FA_{0.67}MA_{0.33}PbI_{2.85}Br_{0.15}$/PCBM/AZO/ITO solar cell. $|E|^2$ is simulated for both illumination from the glass/ITO (bottom) side and ITO/AZO (top) side.

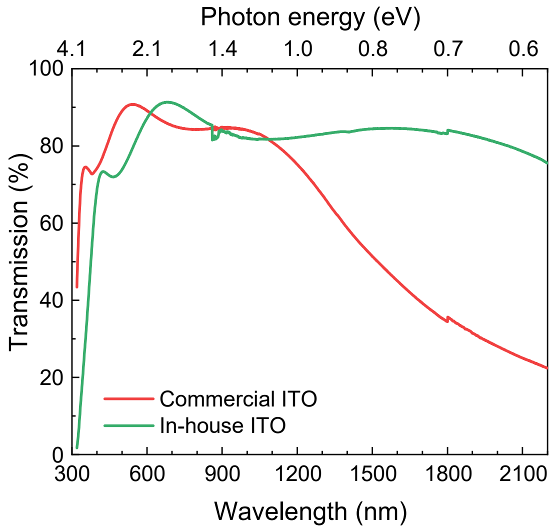

Supplementary Figure 5. UV-vis-NIR transmission spectra of commercial and in-house sputtered ITO, used as bottom and top contact, respectively. The sheet resistances were 17 Ω sq^{-1} and 45 Ω sq^{-1} for the commercial and in-house ITO, respectively.

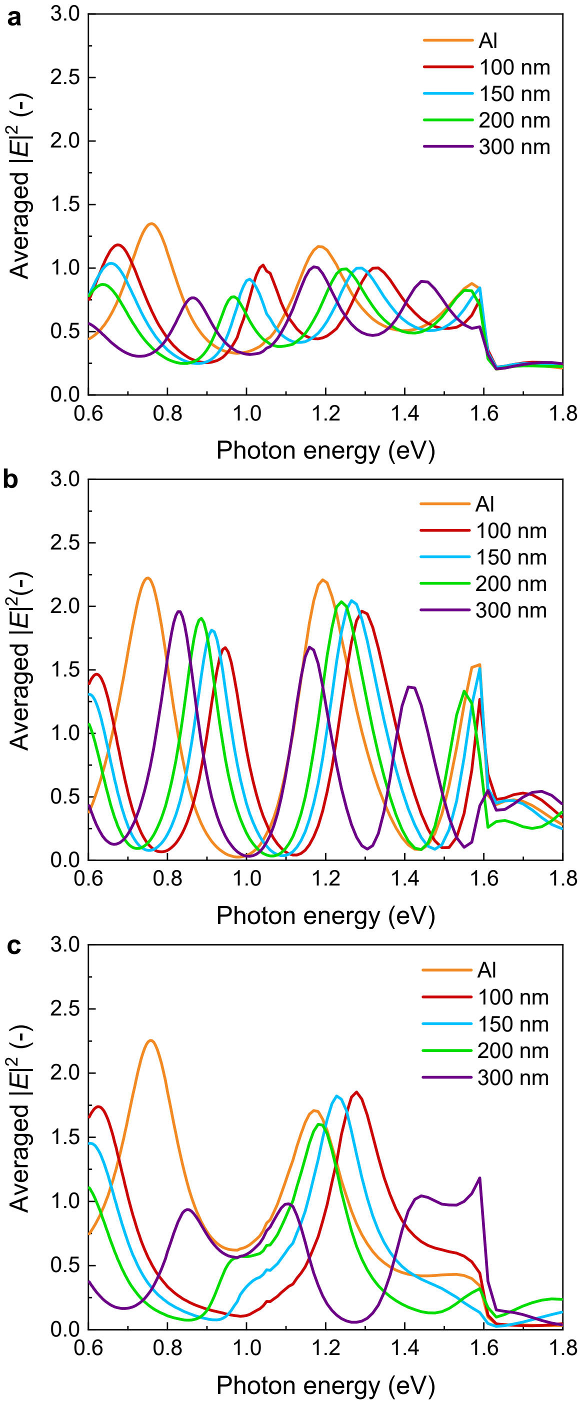

Supplementary Figure 6. Simulated optical electric field $|E|^2$ in p-i-n perovskite solar cells for a glass/ITO/PTAA/$FA_{0.67}MA_{0.33}PbI_{2.85}Br_{0.15}$/PCBM/AZO/Al solar cell and for four glass/ITO/PTAA/$FA_{0.67}MA_{0.33}PbI_{2.85}Br_{0.15}$/PCBM/AZO/ITO/$MgF_2$/Ag devices with MgF_2 optical spacers of different thicknesses as indicated in the legends. **a** $|E|^2$ averaged over the full perovskite layer. **b** $|E|^2$ weighted by a half-normal distribution at the PTAA-perovskite interface. **c** $|E|^2$ weighted by a half-normal distribution at the perovskite-PCBM interface.

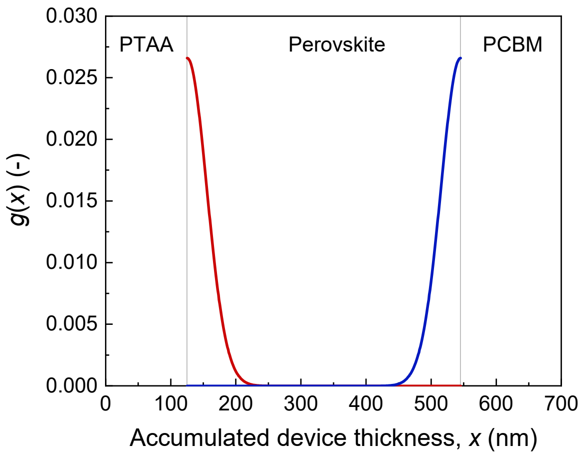

Supplementary Figure 7. Illustration of the half-normal distribution functions $g(x)=\frac{1}{\sigma}\sqrt{\frac{2}{\pi}}\exp\left[-\frac{1}{2}\left(\frac{x-x_i}{\sigma}\right)^2\right]$ used to weigh $|E|^2$ in the perovskite layer near the interfaces with the charge transport layers. Here, x is the depth in the device taken from the glass/ITO interface, x_i = 125 nm for the PTAA-perovskite interface, x_i = 545 nm for perovskite-PCBM interface, and σ = 30 nm. $g(x)$ is set to zero outside the perovskite layer.

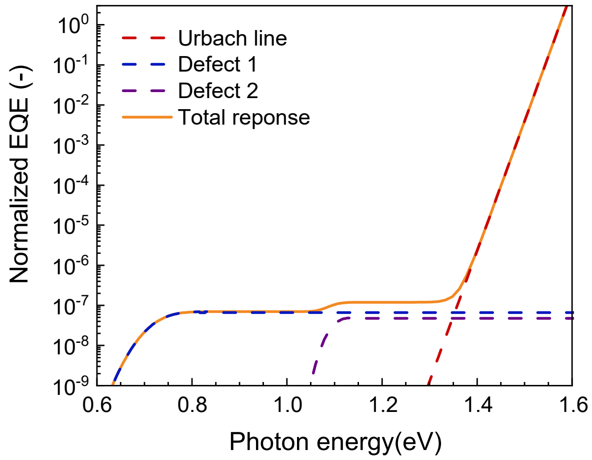

Supplementary Figure 8. Sub-bandgap EQE calculated as the sum of the contributions of two defect functions and a band tail (Urbach) energy. The defect functions are multiplied with the optical electric field functions in Supplementary Fig. 2 to give the spectra shown in Fig. 4 of the main text.

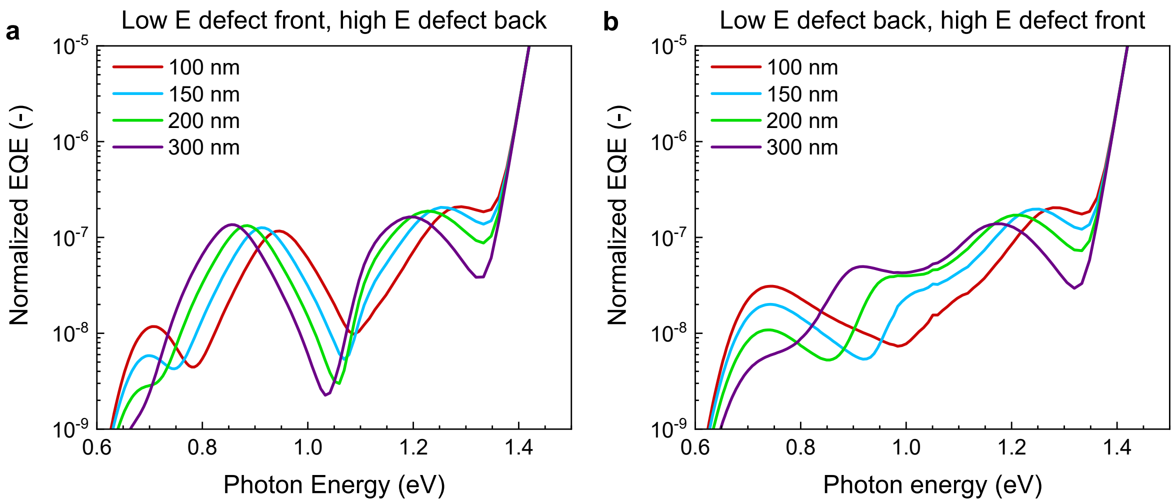

Supplementary Figure 9. Modeled EQE spectra of perovskite solar cells with a transparent back contact and a spacer-mirror on top (glass/ITO/PTAA/$FA_{0.67}MA_{0.33}PbI_{2.85}Br_{0.15}$/ PCBM/ AZO/ITO/$MgF_2$/Ag). **a** Assuming a low photon energy defect at the PTAA-perovskite interface and a high photon energy defect at the perovskite-PCBM interface. **b** Assuming a high photon energy defect at the PTAA-perovskite interface and a low photon energy defect at the perovskite-PCBM interface. In each case an Urbach tail has been added. The legends give the thickness of the MgF_2 optical spacer.

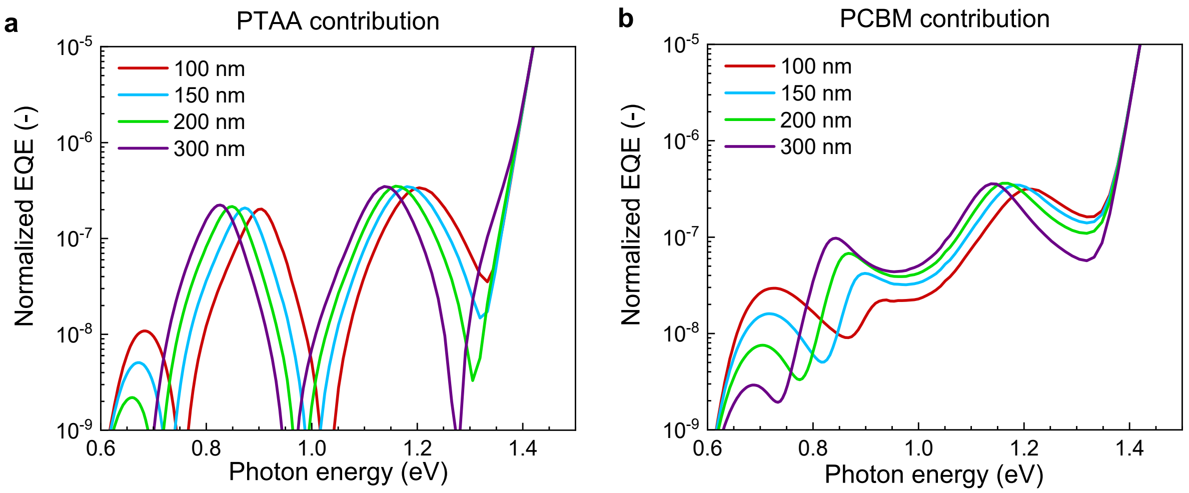

Supplementary Figure 10. Modeled EQE spectra of perovskite solar cells with a transparent back contact and a spacer-mirror on top (glass/ITO/PTAA/$FA_{0.67}MA_{0.33}PbI_{2.85}Br_{0.15}$/PCBM/ AZO/ITO/$MgF_2$/Ag). **a** Assuming defects located in the PTAA hole transport layer cause the sub-bandgap EQE. **b** Assuming defects located in the PCBM electron transport layer cause the sub-bandgap EQE.

Supplementary Note 1

To simulate the sub-bandgap EQEs, shown in Fig. 4, two defect states were assumed that can be excited at photon energies of 0.72 and 1.09 eV and give rise to sub-bandgap photocurrents as described by equation (2) (Supplementary Fig. 4). The parameters used are $\kappa G_{d1} = 7 \times 10^{-8}$, $E_1 = 0.72$ eV, $\sigma_1 = 0.04$ eV for defect 1; and $\kappa G_{d2} = 5 \times 10^{-8}$, $E_2 = 1.09$ eV, $\sigma_2 = 0.04$ eV for defect 2. The summed contributions are then multiplied with the values for $|E|^2$ shown in Supplementary Fig. 2 for the different spatial defect distributions. An exponential band edge as described by equation (1) is then added to the calculated photocurrent. $E_g = 1.574$ eV and $E_u =$ 13.4 meV used in equation (1) were obtained from a fit to the experimental EQE in Fig. 1. In this approach, we assume that the exponential band edge is not affected by optical interference. In fact, optical interference has a small effect as can be seen in Fig. 2b from the slight differences in this region in the shape of exponential tail for the four optical spacers. However, given the relatively minor deviation compared to the significant changes in the sub-bandgap region and the fact that the nature of the exponential band tail might be unrelated to the defect density that gives rise to the sub-bandgap EQE, the contribution of the exponential Urbach contribution is assumed to be constant in the simulations.
