# Supplementary material for: Revealing defective interfaces in perovskite solar cells from highly sensitive sub-bandgap photocurrent spectroscopy using optical cavities
Source: Nat Commun. 2022 Jan 17;13:349. doi: 10.1038/s41467-021-27560-6 (PMC8764070; doi:10.1038/s41467-021-27560-6)
Supplement: Supplementary file 3 — Solar Cells Reporting Summary [file 41467_2021_27560_MOESM3_ESM.pdf]

nature research

Corresponding author(s): Rene Janssen

Solar Cells Reporting Summary

Nature Research wishes to improve the reproducibility of the work that we publish. This form is intended for publication with all accepted papers reporting the characterization of photovoltaic devices and provides structure for consistency and transparency in reporting. Some list items might not apply to an individual manuscript, but all fields must be completed for clarity.

For further information on Nature Research policies, including our data availability policy, see Authors & Referees.

Experimental design

Please check: are the following details reported in the manuscript?

1. Dimensions

Item	Yes	No	Details
Area of the tested solar cells	☒	☐	Cell area 0.09 cm2. see Methods section, device fabrication
Method used to determine the device area	☒	☐	See Methods section, device fabrication

2. Current-voltage characterization

Item	Yes	No	Details
Current density-voltage (J-V) plots in both forward and backward direction	☒	☐	See Supplementary Figure 2a
Voltage scan conditions<br>*For instance: scan direction, speed, dwell times*	☒	☐	See Methods section, device characterization
Test environment<br>*For instance: characterization temperature, in air or in glove box*	☒	☐	See Methods section, device characterization
Protocol for preconditioning of the device before its characterization	☒	☐	No preconditioning was used, see Methods section, device characterization
Stability of the J-V characteristic<br>*Verified with time evolution of the maximum power point or with the photocurrent at maximum power point; see ref. 7 for details.*	☐	☒	Cells were stable during sub-bandgap EQE measurements

3. Hysteresis or any other unusual behaviour

Item	Yes	No	Details
Description of the unusual behaviour observed during the characterization	☒	☐	Minimal hysteresis was found. Mentioned in the main text.
Related experimental data	☒	☐	Supplementary Figure 2a

4. Efficiency

Item	Yes	No	Details
External quantum efficiency (EQE) or incident photons to current efficiency (IPCE)	☒	☐	Supplementary Figure 2b
A comparison between the integrated response under the standard reference spectrum and the response measure under the simulator	☒	☐	Integrated response is mentioned in the Methods section, minimal difference with the current density from the solar simulator. See Table with Supplementary Figure 2b
For tandem solar cells, the bias illumination and bias voltage used for each subcell	☐	☒	No tandem cells are reported.

5. Calibration

Item	Yes	No	Details
Light source and reference cell or sensor used for the characterization	☒	☐	See Methods section, device characterization
Confirmation that the reference cell was calibrated and certified	☒	☐	See Methods section, device characterization

Calculation of spectral mismatch between the reference cell and the devices under test

☐ Yes
☒ No

Short-circuit current densities measured match well with integrated EQE response.

6. Mask/aperture

Size of the mask/aperture used during testing

☒ Yes
☐ No

Mask: 0.0676 cm2, see Methods section, device characterization

Variation of the measured short-circuit current density with the mask/aperture area

☐ Yes
☒ No

Only one aperture was used.

7. Performance certification

Identity of the independent certification laboratory that confirmed the photovoltaic performance

☐ Yes
☒ No

No independent certification of the efficiencies was performed. The efficiecies reported are v in line with expectations and were not the focus of this study.

A copy of any certificate(s)
Provide in Supplementary Information

☐ Yes
☒ No

Not applicable

8. Statistics

Number of solar cells tested

☐ Yes
☒ No

EQE experiments were reproducible.

Statistical analysis of the device performance

☐ Yes
☒ No

EQE experiments were reproducible.

9. Long-term stability analysis

Type of analysis, bias conditions and environmental conditions
For instance: illumination type, temperature, atmosphere humidity, encapsulation method, preconditioning temperature

☐ Yes
☒ No

Stability was not tested.
